# n-Octadecane/Fumed Silica Phase Change Composite as Building Envelope for High Energy Efficiency

**DOI:** 10.3390/nano11030566

**Published:** 2021-02-24

**Authors:** Giang Tien Nguyen, Ha Soo Hwang, Jiyoung Lee, Dong An Cha, In Park

**Affiliations:** 1 Research Institute of Clean Manufacturing System, Korea Institute of Industrial Technology, 89 Yangdaegiro-gil, Ipjang-myeon, Cheonan 31056, Korea; giangnt@kitech.re.kr (G.T.N.); heliocity@kitech.re.kr (H.S.H.); ttoittoijy@kitech.re.kr (J.L.); cdongan@kitech.re.kr (D.A.C.); 2 Korea Institute of Industrial Technology (KITECH) School, University of Science and Technology (UST), 176 Gajeong-dong, Yuseong-gu, Daejeon 34113, Korea; 3 R&D Center, OomphChem Inc., 1223-24 Cheonan-daero, Seobuk-gu, Cheonan 31080, Korea; 4 Department of Chemical and Biomolecular Engineering, Yonsei University, 50 Yonsei-ro, Seodaemun-gu, Seoul 09722, Korea

**Keywords:** fumed silica, phase change material, building envelope, latent heat energy storage, building energy conservation

## Abstract

A novel n-octadecane/fumed silica phase change composite has been prepared as a building envelope with a high content of phase change material and improved energy efficiency. With a high porosity (88 vol%), the fumed silica provided sufficient space to impregnate a high quantity of n-octadecane (70 wt%). The composite exhibited high latent heat storage capacity (155.8 J/g), high crystallization fraction (96.5%), and a melting temperature of 26.76 °C close to that of pure n-octadecane. A 200 accelerated thermal cycle test confirmed good thermal reliability and chemical stability of the phase change composite. The thermal conductivity of n-octadecane was reduced by 34% after impregnation in fumed silica. A phase change composite panel was fabricated and compared to a commercial polystyrene foam panel. When used as the roof of a test room, the phase change composite panel more efficiently retarded heat transfer from a halogen lamp to the room and delayed the increase in the indoor temperature than that by the polystyrene panel. The indoor temperatures of the room with the phase change composite panel roof were 19.8 and 22.9 °C, while those with the polystyrene panel roof were 29.9 and 31.9 °C at 2200 and 9000 s after lamp illumination.

## 1. Introduction

With improved living standards, the rising population, as well as worldwide urbanization, the demand for energy in buildings has continuously increased. Globally, buildings account for up to 40% of total energy consumption, which results in problems such as environmental pollution and energy shortages [1,2]. Energy conservation in buildings, therefore, has become an important issue in energy research. Using phase-change materials (PCMs) as building envelopes is one of the most effective methods for energy conservation and human thermal comfort [3]. The first PCM applications in buildings were for thermal energy storage, and the materials were further developed as components of composites with construction materials such as concrete and cement [1,4]. PCMs can store large quantities of thermal energy in the form of latent heat during their phase transition from solid to liquid, and the temperature of PCMs is constant during this process. Owing to these characteristics, PCMs have also been studied in the form of wallboards [5] or panels [6] to increase the thermal mass of lightweight buildings because they tend to have high temperature variations. During the daytime, the PCMs melt and absorb heat; at night, they solidify and release the absorbed heat [7]. PCM building envelopes can impede the heat flux entering the building from outside, thus lowering the energy consumption for heating, cooling, and ventilation systems [8]. Moreover, some researchers have suggested that building envelopes containing PCMs could be used as thermal insulation materials [8,9].

The selection of PCMs for building envelopes primarily depends on their melting temperatures. Based on the thermal comfort (20 < T < 26 ℃) of human beings [10], PCMs with melting temperatures of 20–32 °C are most suitable [11]. Several potential candidates can be classified as organic PCMs, such as paraffin (C_13_–C_24_) [12], alkanes (n-hexadecane [13], n-octadecane [14]), an eutectic mixture of capric acid and lauric acid [5], fatty acid esters [15], bio-PCMs [16]; and inorganic PCMs such as salt hydrates (CaCl_2_‧6H_2_O [9], LiNO_3_‧3H_2_O [6]). Inorganic PCMs normally present higher latent heat storage capacity than organic PCMs; however, they have many application issues, such as super-cooling, phase segregation, and corrosiveness. Compared to inorganic PCMs, organic PCMs exhibit negligible super-cooling without phase segregation and corrosiveness. Moreover, organic PCMs exhibit lower thermal conductivity than inorganic PCMs [1,4]. Therefore, organic PCMs may show better performance in building applications. However, PCMs generally cannot be directly employed without porous supports or capsules because they tend to leak in the liquid state [17,18].

To solve the leakage problem, the most popular method is the impregnation of PCMs into porous supports to form phase-change composites (PCCs) [19,20]. Owing to the capillary and surface tension forces, the melted or dissolved PCMs are firmly confined in the porous network of the support [21]. For practical applications in building envelope materials, the porous support should be low-cost, abundant, and non-toxic, as well as exhibit high PCM adsorption capacity and low thermal conductivity. Aerogels have emerged as one of the most promising porous materials because of their large pore volume, high porosity (>90 vol%), and ultra-low thermal conductivity (<0.035 W/m K). However, their production requires a multistep process followed by supercritical drying, making them uneconomical for large-scale applications [3,22]. Fumed silica (FS), an inexpensive and commercialized siliceous material, possesses a similar framework structure of nanosized silica particles fused into short chains [23]. Thus, it exhibits low weight and thermal conductivity (<0.05 W/m K), which are comparable to those of silica aerogel [3]. Given these advantages, FS has been widely applied as a conventional insulation material [3,24]. Furthermore, FS possesses rich nanopores (~100 nm) with a high surface area and high porosity (ca. 90%), and can impregnate and confine a large quantity of PCM for PCCs. Chen et al. [25] reported that hydrophobic FS can hold up to 85 wt% dodecane when applied for cold energy storage. Consequently, FS is a favorable candidate for the development of novel PCCs with high energy storage capacity and low thermal conductivity. 

Fumed silica materials are commercially available with various nano-structures, porosities, and surface properties, i.e., hydrophobic and hydrophilic surface. Qu et al. [8] applied a paraffin/hydrophobic FS PCC for building to a concrete block for energy conservation. However, the hydrophobic FS, which requires a surface modification with expensive silane agents and organic solvents, is not preferable for large-scale applications as well as environments. Alternatively, Peng et al. [26] employed a small amount of hydrophilic FS (≤4%) as an additive to improve the supercooling issue, thermal reliability, and thermal stability of an inorganic Na_2_HPO_4_‧12H_2_O PCM. Fu et al. [27] characterized a sodium acetate trihydrate-urea/hydrophilic FS PCC for a floor heating system. However, no report has investigated a PCC with hydrophilic FS as a porous matrix for building envelope applications, to the best of our knowledge.

Based on the above discussion, we herein report an n-octadecane/FS PCC as a novel building envelope for energy saving and thermal comfort. A low-cost hydrophilic FS was adopted as a supporting matrix. n-Octadecane was chosen as the PCM owing to its suitable melting point (~28 °C) close to room temperature and high latent heat storage capacity (~250 J/g). n-Octadecane was impregnated into the FS by an evaporative solution impregnation method. The microstructure, morphology, chemical compatibility, crystallinity, thermal reliability, thermal stability, phase change behaviors, and thermal conductivity of the PCC were thoroughly characterized. A large amount of PCM (70 wt%) was impregnated in the FS without leakage, and with a high heat storage capacity (155.8 J/g), and reduced thermal conductivity. In addition, the thermal performance of the PCC was evaluated and compared to that of a commercialized polystyrene (PS) foam panel, when constructed as the roof of a test room. The PCC could reduce the indoor peak temperature and delay the indoor temperature rise, compared to the PS material.

## 2. Materials and Methods

### 2.1. Materials 

FS (Aerosil 200) was obtained from Evonik Korea (Seoul, Korea). n-Octadecane (99%) was obtained from Alfa Aesar (Ward Hill, MA, US). n-Hexane (95%) was purchased from Samchun Chemical (Seoul, Korea). PS foam panels with a density of 20 kg/m^3^ were obtained from ChunwooEPS (Changnyeong, South Korea).

### 2.2. Preparation of n-Octadecane/Fumed Silica (FS) Phase-Change Composites (PCC)

The FS was heated at 200 °C for 24 h to remove physically adsorbed water. Subsequently, n-octadecane/FS composites were prepared by an evaporative solution impregnation method [28] with various contents of n-octadecane (60−75 wt%). Typically, a predetermined amount of n-octadecane was dissolved in hexane, and a pre-calculated amount of FS was introduced into the above solution. The mixture was magnetically stirred for 5 h at 25 °C and subsequently heated to 60 °C until the solvent evaporated. The as-obtained white powder was dried in an oven at 60 °C overnight for complete solvent evaporation.

### 2.3. Characterization Methods

Scanning electron microscopy (SEM) was performed using a JSM 6701 instrument (JEOL, Tokyo, Japan) with a beam energy of 5 kV. The nitrogen adsorption-desorption isotherms were recorded using a BELSORP-Max instrument (MicrotracBel, Osaka, Japan) at the temperature of liquid nitrogen (−196 °C). The surface area was obtained using the Brunauer–Emmett–Teller theory. The pore size distribution was calculated from the adsorption branch using the non-local density functional theory. The porosities of the materials were analyzed by mercury intrusion porosimetry, using a Microactive Autopore V 9600 (Micromeritics, Norcross, GA, US). The measurements were conducted at 20 °C in the pressure range of 0.1 to 61,000 psi.

The chemical compositions of the materials were evaluated using a Nicolet 6700 Fourier transform infrared (FT-IR) spectrometer (Thermo Scientific, Waltham, MA, US) in the transmittance mode.

The crystallinity of the materials was characterized by X-ray diffraction (XRD) using a powder X-ray diffractometer (Rigaku Miniflex, Tokyo, Japan) with Cu-Kα radiation. The testing current and voltage were 15 mA and 40 kV, respectively. The scanning rate was 5°/min in the 2*θ* range 5–50°.

The phase change properties of the materials were investigated using a DSC 4000 differential scanning calorimeter (Perkin Elmer, Waltham, MA, US). The measurements were performed at a temperature ramp rate of 5 °C/min with N_2_ purge at 20 mL/min and a temperature range of −10–45 °C. The phase change temperatures were regarded as the onset temperatures. Each measurement was performed for two cycles, and the first cycle was excluded to eliminate the thermal history. 

The thermal stabilities of the materials were tested by thermogravimetric (TG) analysis using a 4000 thermogravimetric analyzer (Perkin Elmer, Waltham, MA, US). The temperature range was 30–500 °C, accompanied by a ramp rate of 10 °C/min and N_2_ purge of 20 mL/min. The decomposition temperatures were calculated at the onset temperatures.

The thermal reliability of the composite was examined by a 200 thermal cycle test (1 °C ↔ 60 °C) under ambient atmosphere, using two temperature-controlled water baths. The temperature of the cool bath (1 °C) was controlled by ice, while that of the hot bath (60 °C) was controlled by a hot plate. The composite (1 g) was placed in a glass vial and then shifted between the two baths, where it was kept for 5 min.

Thermal conductivities were determined by the transient plane source method at room temperature, using a thermal constant analyzer (TPS 3500, Hot-Disk AB, Goteborg, Sweden). Subsequently, 11.6 g of PCCs was compressed into two round blocks with a diameter of 30 mm and a depth of 10 mm with a homemade mold. For comparison, the pure PCM was melted and poured into the mold to obtain two round blocks of the same size as the PCC blocks. The thermal conductivity was measured for four cycles to obtain an average value.

### 2.4. Evaluation of the Thermal Performance of n-Octadecane/FS PCC

To evaluate the smoothing performance of the temperature fluctuation in a lab scale, the thermal performances of PCC panels in previous reports were tested in small test rooms. A polystyrene panel or a gypsum panel, conventionally employed as building envelopes, were used in small room tests [9,29]. We hereby follow the methodology to evaluate the thermal performance of our n-octadecane/FS PCC.

#### 2.4.1. Fabrication of PCC Panel and Polystyrene Panel

**Figure 1:**
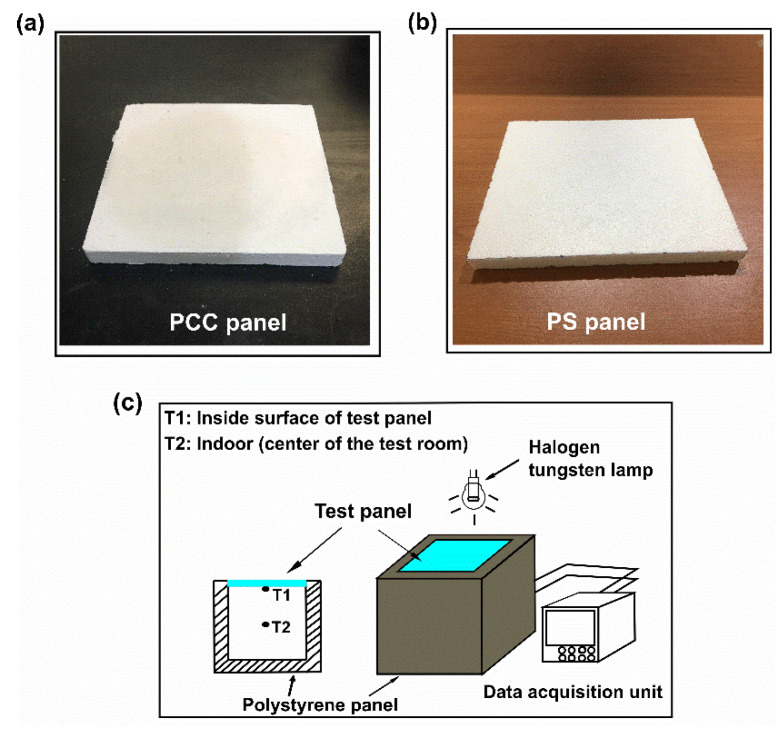
Optical images of (**a**) phase-change composite (PCC) panel, and (**b**) polystyrene (PS) panel, and (**c**) schematic diagram of the experimental setup for examining the temperature variations in a test room.

A 70 wt% n-octadecane/FS composite was fabricated into a panel (Figure 1a), using a homemade mold and compressor. The PCC panel had dimensions of 200 × 200 × 20 mm. For comparison, a PS panel was cut to the same size as that of the PCC panel (Figure 1b). 

#### 2.4.2. Thermal Performance Evaluation

The experimental setup for examining temperature variations in a test room is shown in Figure 1c. The test room (200 × 200 × 200 mm) was built with five PS panels. One panel (300 × 300 × 50 mm) was employed as the base, and four panels (250 × 220 × 50 mm) were used as the walls. The as-fabricated PCC and PS panels were utilized as roofs in the room. A halogen tungsten lamp (Philip PAR38, Amsterdam, The Netherlands) was employed as the solar simulator and hung 130 mm above the roof of the test room. The ends of a T-type thermocouple connected to a data acquisition unit (Yokogawa, MV200, Tokyo, Japan) were positioned at the inner surface of the test panel and the center of the room. The test began as the lamp was switched on, and the temperature fluctuations at the two positions were recorded. After 12,600 s, the lamp was switched off and the temperature variations during cooling were measured.

## 3. Results and Discussion

### 3.1. Characterization of n-Octadecane/FS PCCs

**Figure 2:**
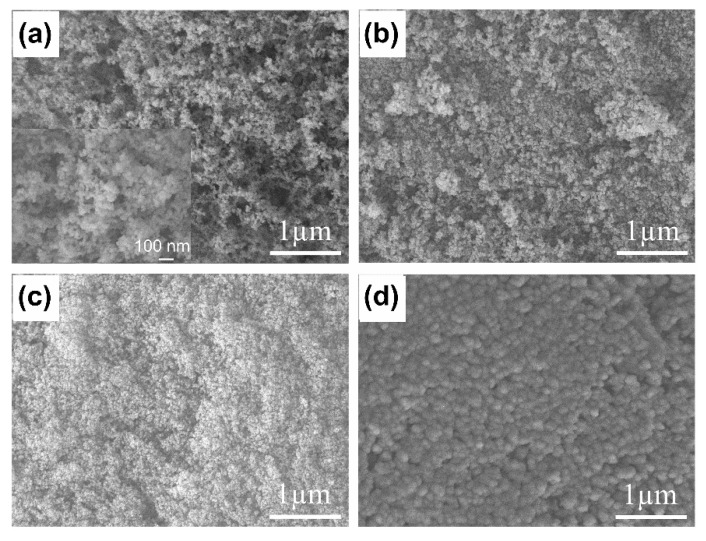
Scanning electron microscopy (SEM) images of (**a**) fumed silica (FS), and (**b**–**d**) n-octadecane/FS composites with various n-octadecane mass fractions: (**b**) 60 wt%, (**c**) 70 wt%, and (**d**) 75 wt%.

**Figure 3:**
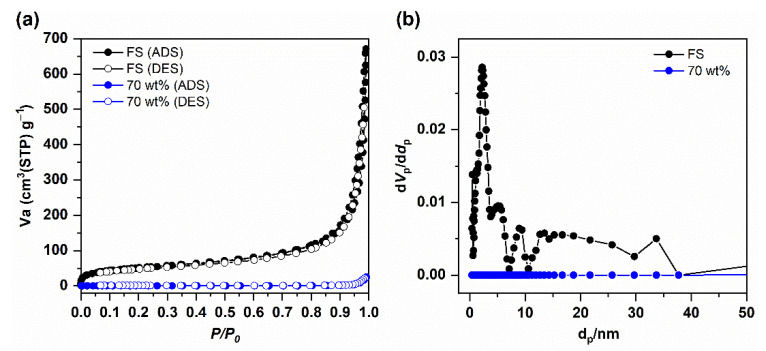
(**a**) N_2_ sorption isotherms of FS and the 70 wt% composite, and (**b**) pore size distributions of FS and the 70 wt% composite.

The SEM image of FS (Aerosil 200, Evonik Korea, Seoul, Korea) in Figure 2a shows that the nanoparticles formed an interconnected porous network with a high proportion of macropores, allowing impregnation of the PCM. The N_2_ sorption isotherm of the FS was measured, as shown in Figure 3a. A type II isotherm was obtained, but the micropore volume of the FS was not well developed. The interconnected silica nanoparticles formed nanosized pores in the range of 50–150 nm [23]. Due to the limited pore size distribution obtained from the isotherm, mercury intrusion porosimetry was performed (Figure S1). The pore size distributions show that the FS possesses a wide range of meso- and macroporosities. The porosity obtained from mercury intrusion was 88 vol%. The pore volumes and surface areas were 1.0 cm^3^/g and 185 m^2^/g, respectively, as calculated from the adsorption branch of the N_2_ sorption isotherm and 17 cm^3^/g and 205 m^2^/g, respectively, as calculated from mercury intrusion porosimetry.

The SEM images of the PCCs with various n-octadecane mass fractions are shown in Figure 2b–d. The porous framework of the FS in the 60 wt% sample (Figure 2b) was partially occupied by the PCM. When the n-octadecane content increased to 70 wt% (Figure 2c), the pores of the FS were more occupied, indicating the successful impregnation of n-octadecane into the FS. The N_2_ sorption isotherm of the 70 wt% composite with a reduced uptake of nitrogen (Figure 3a) and the disappearance of pores in its corresponding pore size distribution (Figure 3b) further verified the impregnation of the PCM in the mesopores of the FS. The mercury porosimetric curve of 70 wt% PCC (Figure S1) showed that pores sized less than 300 nm were filled and the larger pores were considerably filled, indicating that the PCM covered the surface of the SiO_2_ nanoparticles. The SEM image of the sample containing 75 wt% of PCM (Figure 2d) showed the absence of small pores and an excess amount of bulk PCM covering the silica particles.

To evaluate the anti-leakage ability of the prepared composites, a leakage test was conducted. The pure n-octadecane and the composites were placed on filter papers and kept in an oven at 50 °C (~20 °C higher than the melting point of n-octadecane) for 60 min. The results are shown in Figure S2. While n-octadecane was completely liquidized, the composites with 60 wt% and 70 wt% PCM were maintained without leakage owing to the capillary and surface tension forces in the FS. However, the sample with 75 wt% of PCM showed slight leakage; thus, it could not be used as a PCC. TG analysis was conducted to further confirm the anti-leakage ability of the 70 wt% composite, as shown in Figure S3. No significant difference in the weight losses could be observed between the samples before and after the leakage test, demonstrating that the PCCs were capable of maintaining good shape stability with up to 70 wt% of n-octadecane. A higher content of the PCM would benefit the thermal storage capacity of the composite. Therefore, 70 wt% PCM was selected as the optimal mass fraction for the PCC. 

**Figure 4:**
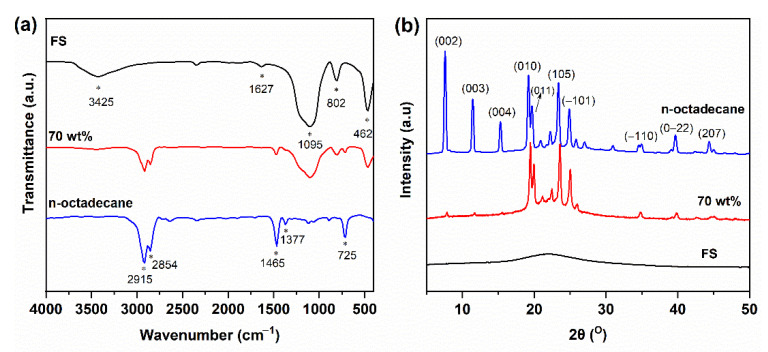
(**a**) Fourier transform infrared (FT-IR) spectra of FS, n-octadecane, and 70 wt% PCC. (**b**) X-ray diffraction (XRD) patterns of FS, n-octadecane, and 70 wt% PCC.

The chemical compatibility of the composites was evaluated by FT-IR spectroscopy, as shown in Figure 4. In the IR spectrum of FS, the broad band centered at 3425 cm^−1^ was due to the overlapping stretching vibration of surface silanol groups (−Si−OH) and surface-adsorbed water molecules [30]. The adsorbed water was observed with a bending vibration at 1627 cm^−1^. Meanwhile, the bands at 1095, 802, and 462 cm^−1^ were assigned to the Si–O–Si asymmetric stretching vibration, symmetric stretching vibration, and bending mode, respectively. In the case of n-octadecane, the bands at 2915 and 2854 cm^−1^ were attributed to the stretching vibration of −C−H bonds, while those at 1465 and 1377 cm^−1^ were assigned to the −C−H bending vibration. The peak at 725 cm^−1^ belonged to the in-plane rocking mode of the –CH_2_− groups. For the 70 wt% PCC, the characteristic peaks of n-octadecane overlapped with those of the SiO_2_ matrix. Furthermore, no additional peaks were observed. These results indicated that the composite components were physically compounded with no chemical reactions, and thus possessed good chemical compatibility.

The crystallization behaviors of the pristine materials and their composites were characterized by XRD, as shown in Figure 4b. The FS showed a broad peak in the 2*θ* range of 15–30°, typical of an amorphous SiO_2_ structure. The pure n-octadecane was characterized by a mixture of two crystal phases [31]: the peaks at 2*θ* = 7.5, 11.5, 15.3, 39.8, and 44.7° were attributed to the (002), (003), (004), (0−22), and (207) planes of the α-crystal form, and the peaks at 2*θ* = 19.2, 19.8, 23.4, 24.9, and 35.2° were assigned to the (011), (011), (105), (111), and (−110) planes of the β-crystal form, respectively. The 70 wt% PCC showed mixed patterns of PCM and SiO_2_, further confirming that the composite components were physically combined without chemical reactions. The crystallinity of the PCC is discussed in Section 3.2. 

### 3.2. Phase Change Properties of n-Octadecane/FS PCC

**Figure 5:**
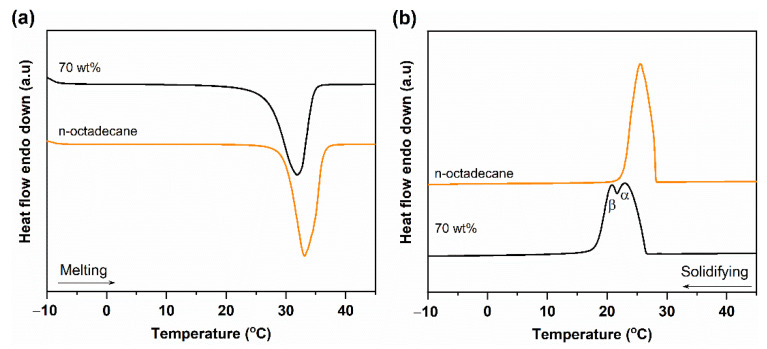
(**a**) Melting and (**b**) solidification differential scanning calorimeter (DSC) curves of pure n-octadecane and the 70 wt% PCC.

The phase change properties of pure n-octadecane and PCC were investigated by DSC, as shown in Figure 5. The melting/solidifying temperatures (T_M_/T_S_) and the melting/solidifying phase change enthalpies (ΔH_M_/ΔH_S_) obtained from the DSC curves are summarized in Table 1. The pure n-octadecane showed a single endothermic peak (28.81 ± 0.12 °C) during melting and exothermic peak (28.21 ± 0.14 °C) during solidification. Both the endothermic and exothermic peaks were asymmetric, suggesting the presence of more than one crystalline phase. Indeed, the n-octadecane crystal consisted of two crystalline phases, α- and β-, resulting from heterogeneous and homogeneous nucleations, respectively, and the β-crystal phase was dominant [31]. Under certain conditions, the two crystalline phases showed very close crystallizing points, thus overlapping with each other. For the 70 wt% PCC, T_M_ and Ts were 26.76 ± 0.10 °C and 26.69 ± 0.37 °C, respectively, slightly lower than those of pure n-octadecane. The decreased phase change temperature of n-octadecane in the FS was a typical phenomenon due to confinement effects and has been observed in other PCCs [32]. During melting, the PCC showed one peak (Figure 5a), and its solidifying process was quite different as compared to the pristine PCM (Figure 5b). The α- and β-crystalline phases were separated due to a shift in the β-phase peak to a lower temperature than that of pure n-octadecane (see details in Table 1). As n-octadecane was confined in the porous network of the FS, the homogeneous nucleation of the β-phase was limited, causing a shift in the crystallization temperature. Simultaneously, the α-crystal peak exhibited a higher intensity than the β-crystal peak, revealing that heterogeneous nucleation was dominant in the PCC. 

As for the phase change enthalpies, pure n-octadecane exhibited ΔH_M_ and ΔH_S_ of 230.5 ± 6.0 J/g and 229.4 ± 3.7 J/g, respectively. They are in good agreement with data of other literature in which the ΔH_M_ and ΔH_S_ of pure n-octadecane were between 214.6 and 236.6 J/g [14,33]. The 70 wt% PCC exhibited ΔH_M_ and ΔH_S_ of 155.8 ± 2.5 J/g and 154.7 ± 1.2 J/g, respectively. For a PCC, the crystallinity could be evaluated by calculating the crystallization fraction (F), as described in the following equation (Equation (1)) [34];

(1) $$F=\frac{\Delta H_{M,PCC}+\Delta H_{S,PCC}}{(\Delta H_{M,PCM}+\Delta H_{S,PCM})\times x}\times 100\%$$

where ΔH_M,PCC_ and ΔH_S,PCC_ are the melting and solidifying enthalpies of the PCC, respectively, and ΔH_M,PCM_ and ΔH_S,PCM_ are the melting and solidifying enthalpies of the pure PCM, respectively. x is the relative mass proportion of the PCM in the composite. The crystallized fraction of the 70 wt% n-octadecane/FS PCC was calculated to be 96.5 ± 3.4% according to Table 1, suggesting that confinement in the porous framework of the FS did not affect the crystallinity of n-octadecane. As a result, the PCC could exhibit a high crystallized fraction, resulting in a high latent heat storage capacity (155.8 ± 2.5 J/g).

The PCM content and thermal energy storage capacity of the n-octadecane/FS PCC were compared to those of previously reported PCCs that had melting points suitable for building applications, as shown in Table 2. While the n-octadecane/FS PCC in this work had a PCM content of 70 wt%, most of the reported PCCs exhibited a lower PCM content (≤60 wt%) owing to the low impregnation capacity of their porous supports. As shown in Table 2, exfoliated graphite exhibited 80 wt% loading of fatty acid ester PCCs. In comparison, the n-octadecane/FS composite in this work possessed excellent thermal energy storage capacity. 

### 3.3. Thermal Stability of n-Octadecane/FS PCC

**Figure 6:**
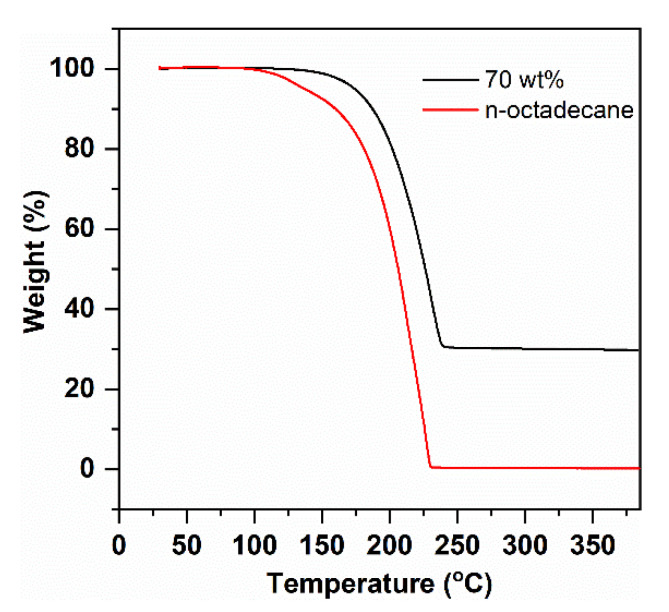
Thermogravimetric (TG) curves of pure n-octadecane and 70 wt% n-octadecane/FS PCC.

Thermal stability is an important property for building applications of PCCs. Herein, the thermal stability was characterized by TG analysis, as shown in Figure 6. The pure PCM presented onset and endset decomposition temperatures of 168 and 230 °C due to the volatilization of n-octadecane, while the as-prepared PCC exhibited values of 192 and 240 °C. The PCC showed improved thermal stability over the pure PCM; this was ascribed to the interfacial interactions (capillary and surface tension forces) between the FS and PCM, which could inhibit the spilling of the PCM out of the FS porous network, thus improving the thermal stability of the PCC [38,39]. The onset decomposition temperature of the PCC (192 °C) was higher than its targeted working temperature (ambient temperature). Therefore, the n-octadecane/FS composite possesses high thermal stability for building envelope applications. 

### 3.4. Thermal Reliability and Chemical Stability of n-Octadecane/FS PCC

**Figure 7:**
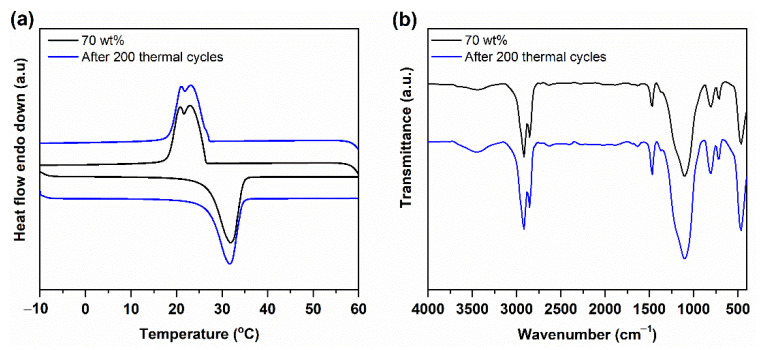
(**a**) DSC curves and (**b**) FT-IR spectra of the 70 wt% n-octadecane/FS PCC before and after 200 thermal cycles.

To determine the long-term working ability of a PCC, its phase change temperature and enthalpy after 200 thermal cycles were recorded. In previous works, the thermal reliability of the PCC was tested using DSC under an inert atmosphere [37]. In this work, to evaluate the thermal reliability of the PCC under practical conditions, a 200 cycle test was conducted under an ambient atmosphere using a temperature-controlled water bath (1 °C ↔ 60 °C). The DSC curves and the detailed phase change properties of the samples after the test are shown in Figure 7a and Table 1, respectively. Compared to the as-prepared composite, the composite subjected to multiple thermal cycles exhibited a comparable DSC curve and phase change temperature. In addition, the phase-change enthalpies were almost unchanged (Table 1). To confirm the chemical stability of the PCC after the thermal cycle test, FT-IR spectra were measured (Figure 7b). No remarkable difference in the shape, intensity, and wavenumber of the absorbed bands could be detected compared to those of the original one, indicating that the PCC had good chemical stability after multiple thermal cycles. All the above results demonstrate that the PCC possesses excellent thermal reliability and chemical stability for long-term utilization.

### 3.5. Thermal Conductivity of n-Octadecane/FS PCC

**Figure 8:**
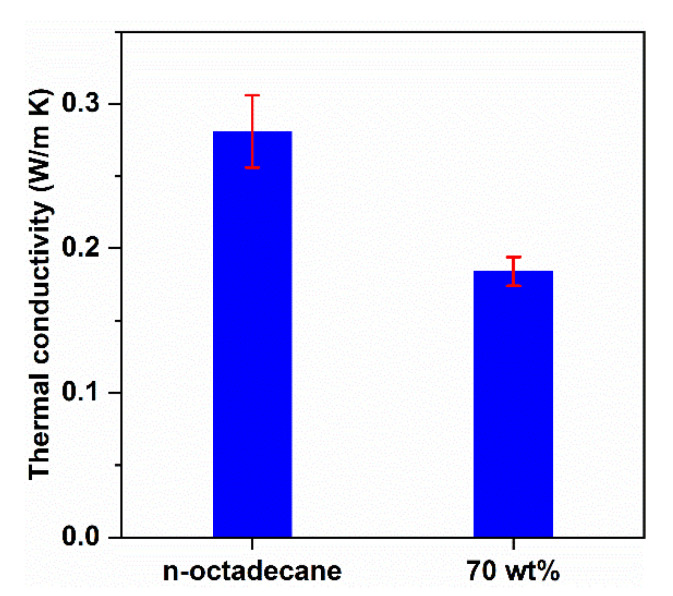
Thermal conductivities of pure n-octadecane and the 70 wt% n-octadecane/FS PCC.

Thermal conductivity is one of the crucial parameters affecting the thermal transfer rate of building envelope materials. In this work, the thermal conductivities were measured using the transient plane source method at room temperature. As shown in Figure 8, the thermal conductivity of n-octadecane was 0.281 ± 0.025 W/m K, which is consistent with previous reports showing that the thermal conductivity of pure n-octadecane is between 0.12 and 0.4 W/m K [40]. Meanwhile, the thermal conductivity of PCC was 0.184 ± 0.010 W/m K. Apparently, the PCC showed a thermal conductivity approximately 34% lower than that of pure n-octadecane. The low thermal conductivity (~0.05 W/m K) of the FS is attributed to the absence of a convection factor because the pore size is of the same order as the mean free path (70 nm) of free air at atmospheric pressure [23]. After the impregnation of n-octadecane, the mesopores of the FS were filled with the PCM (Figure 3b), but macropores larger than 300 nm were partially retained (Figure S1 and Figure 2c). Assuming that 100% of the FS pores are filled with n-octadecane, the theoretical content of the PCM would be 95 wt%, calculated with 88 vol% porosity of the FS. The thermal conductivity of the composite was intermediate to those of the two components. The lower conductivity of the PCC than that of the pristine PCM is a positive parameter, which retards the heat transfer through the PCC.

### 3.6. Thermal Performance of PCC Panel

#### 3.6.1. Properties of PCC Panel

Some thermophysical properties of the PCC and PS panels were measured, as shown in Table 3. The PS panel possessed zero latent heat at ambient temperature, a very low density of 20 ± 0.5 kg/m^3^, and thermal conductivity of 0.045 ± 0.007 W/m K. In comparison with the PS panel, the PCC panel exhibited a latent heat of 155.8 ± 2.5 J/g, revealing its excellent ability to store thermal energy. The PCC panel had a density of 700 ± 18 kg/m^3^, which is 35-fold higher than that of the PS panel. Although the thermal conductivity of the PCC panel was 0.184 ± 0.010 W/m K, approximately 4 times higher than that of the PS panel, it was still at a lower level. 

#### 3.6.2. Thermal Performance of the PCC Panel in a Test Room

**Figure 9:**
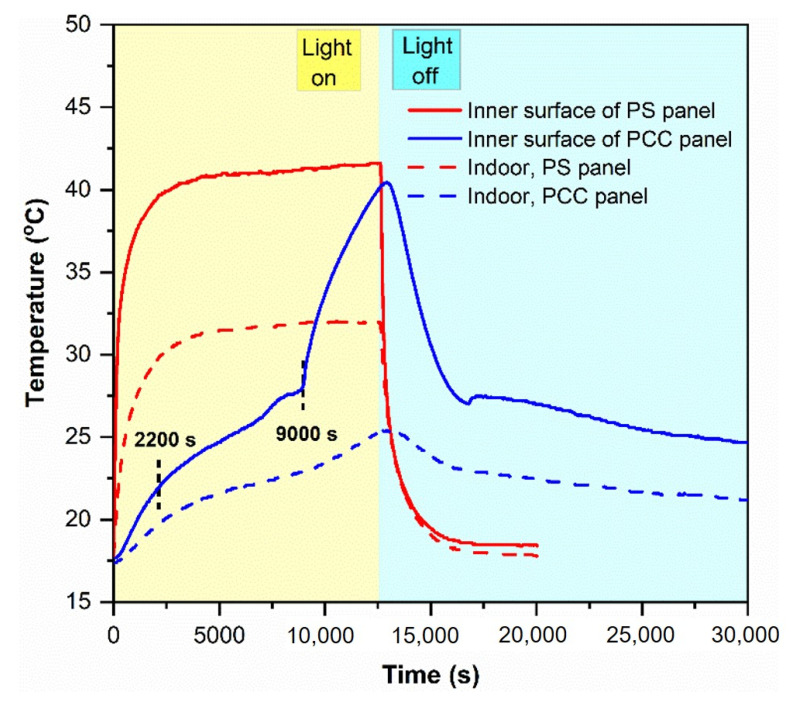
Temperature variations at different locations of the test room integrated with different panels.

To investigate the thermal performance of the PCC panel in a test room in comparison to the commercialized PS panel, the two panels were utilized as the roofs of the test room. A halogen tungsten lamp was used as the solar simulator. During the tests, the temperature variations at the inner surface of the test panels and the center of the test room (indoor) were recorded (see Figure 1 for details). The results of these tests are shown in Figure 9. Based on the tangential method, the temperature rising curve at the inner surface of the PCC panel during illumination can be divided into three steps: 0–2200 s, 2200–9000 s, and 9000–12,600 s. The first step (0–2200 s) and the last step (9000–12,600 s) showed a temperature rise before and after the melting of the PCC, respectively, and were developed by the absorption of sensible heat. The middle step (2200–9000 s) presented the temperature rise during the melting of the PCC, developed by both sensible heat and latent heat absorptions. The slope of the middle step was relatively lower than those of the other two steps. A PCM generally has higher latent heat than the sensible heat. While the sensible heat directed the first and last steps, the latent heat governed the second step, thus leading to the slow temperature rise of the second step. As a result, the inner surface of the PCC panel maintained a low temperature (<27 °C) until 9000 s. In contrast, the temperature at the inner surface of the PS panel rapidly increased to 39.4 °C after 2000 s and reached a peak at approximately 42 °C after 4000 s.

The indoor temperature slightly increased from room temperature to 19.6 °C with the PCC panel roof, while it rose to 29.6 °C with the PS panel roof after 2000 s of illumination. The indoor temperature peaked at approximately 31.5 °C after 4000 s with the PS panel roof, and at ~25 °C after 12,600 s with the PCC panel roof. The PCC panel played a role in reducing the indoor peak temperature and delaying the indoor temperature rise. The PCC panel could decrease the temperature swings and maintain the room temperature closer to a level suitable for human comfort than the PS panel. Although the PCC panel had a higher thermal conductivity than the PS panel, it effectively retarded heat transfer from the illumination of the halogen lamp to the room and maintained the room temperature within the human comfort range. 

## 4. Conclusions

A novel phase change composite, consisting n-octadecane as a PCM and low-cost fumed silica as a supporting material, was prepared by the evaporative solution impregnation method. FS exhibited an interconnected porous structure with high porosity (88 vol%), supplying sufficient space to impregnate n-octadecane, which was firmly confined in pores by capillary and surface tension forces. The FS had a high adsorption capacity for n-octadecane (70 wt%), and the latent heat storage capacity of the PCC was up to 155.8 J/g, superior to previously reported PCCs. The melting temperature of the PCC was 26.76 °C, close to those of the pure n-octadecane (28.21 °C). The thermal stability of n-octadecane improved after its incorporation into FS. Good thermal reliability and chemical stability of the PCC were also proved by a 200 accelerated thermal cycle test. The thermal conductivity of the PCC was only 0.184 W/m K which was reduced by 34% compared to that of pure n-octadecane. The PCC was fabricated into a panel to evaluate its thermal performance in a test room and compared to a commercialized PS panel. The two panels were employed as roofs in a test room. It was found that the PCC panel could reduce the indoor peak temperature and delay the heat flux entering the test room, compared to the PS panel. With the low-cost and aforementioned excellent thermal performance, the n-octadecane/FS PCC has potential applications as a building envelope for energy conservation. For future work, the PCC panel should be applied in an actual building to evaluate the practical thermal performance, heat storage stability, and mechanical durability. 

## Figures and Tables

**Table 1:** Phase change properties of n-octadecane and the 70 wt% PCCs.

Sample	T_M_ (°C)	ΔH_M_ (J/g)	T_S,α_ (°C)	Ts,_β_ (°C)	ΔH_S_ (J/g)	F (%)
n-octadecane	28.81 ± 0.12	230.5 ± 6.0	28.21 ± 0.14		229.4 ± 3.7	100
70 wt%	26.76 ± 0.10	155.8 ± 2.5	26.69 ± 0.37	22.09 ± 0.34	154.7 ± 1.2	96.5 ± 3.4
70 wt% after 200 thermal cycles	26.61 ± 0.24	154.2 ± 4.1	27.07 ± 0.17	22.23 ± 0.22	153.2 ± 4.0	95.5 ± 4.6

**Table 2:** Comparison of phase-change material (PCM) content and latent heat storage capacity of reported PCCs and the n-octadecane/FS PCC.

Phase-Change Composite	PCM Content (wt%)	T_M_ (°C)	ΔH_M_ (J/g)	Ref
Fatty acid ester/exfoliated graphite	80	26.9	77.6	[15]
CaCl_2_‧6H_2_O/expanded perlite	55	27.4	87.4	[9]
n-Dodecanol/SiO_2_ nano-shell	55.5	21.0	116.7	[35]
n-Hexadecane/xGnP	48.8	16–25	96.4	[13]
n-Hexadecane/diatomite	47	23.7	120.1	[36]
n-Octadecane/porous TiO_2_	50	27.9	85.8	[37]
n-Octadecane/activated carbon	43.4	-	101.8	[29]
n-Octadecane/PMMA	60	32.6	125.4	[34]
n-Octadecane /SiO_2_ micro-shell	66.2	23.3	142.2	[33]
n-Octadecane/FS	70	26.7	155.8	This work

**Table 3:** Thermophysical properties of PCM panel and PS panel.

Material	Latent Heat (J/g)	Density (kg/m^3^)	Thermal Conductivity (W/m K)
PS panel	0	20 ± 0.5	0.045 ± 0.007
PCC panel	155.8 ± 2.5	700 ± 18	0.184 ± 0.010

## Supplementary Materials

The following are available online at https://www.mdpi.com/2079-4991/11/3/566/s1, Figure S1: Mercury porosimetry intrusion curves of the pristine FS and the 70 wt% n-octadecane/FS PCC, Figure S2: Photographs of pure n-octadecane and PCCs with various n-octadecane mass fractions from the leakage test, Figure S3: Thermogravimetric analysis (TGA) curves of the 70 wt% n-octadecane/FS PCC before and after the leakage test.

## Nomenclature

PCM	Phase change material
PCC	Phase change composite
FS	Fumed silica
PS	Polystyrene
T_M_	Melting temperature (°C)
T_S_	Solidifying temperature (°C)
T_S,α_	Solidifying temperature of α crystallization phase (°C)
T_S,β_	Solidifying temperature of β crystallization phase (°C)
ΔH_M_	Melting phase change enthalpy (J/g)
ΔH_S_	Solidifying phase change enthalpy (J/g)
F	Crystallization fraction (%)
ΔH_M,PCC_	Melting phase change enthalpy of phase change composite (J/g)
ΔH_S,PCC_	Solidifying phase change enthalpy of phase change composite (J/g)
ΔH_M,PCM_	Melting phase change enthalpy of pure phase change material (J/g)
ΔH_S,PCM_	Solidifying phase change enthalpy of pure phase change material (J/g)
x	Relative mass proportion of the PCM in the composite
SEM	Scanning electron microscopy
FT-IR	Fourier transform infrared spectroscopy
DSC	Differential scanning calorimetry
TGA	Thermogravimetric analysis
