# Supplementary material for: n-Octadecane/Fumed Silica Phase Change Composite as Building Envelope for High Energy Efficiency
Source: Nanomaterials (Basel). 2021 Feb 24;11(3):566. doi: 10.3390/nano11030566 (PMC7996136; doi:10.3390/nano11030566)
Supplement: Supplementary file 1 [file nanomaterials-11-00566-s001.pdf]

Supplementary Material

n-Octadecane/Fumed Silica Phase Change Composite as Building Envelope for High Energy Efficiency

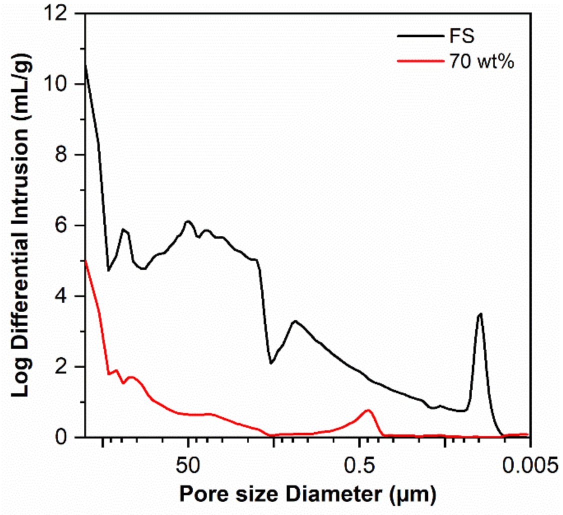

Figure S1. Mercury porosimetry intrusion curves of the pristine FS and the 70 wt% n-octadecane/FS PCC.

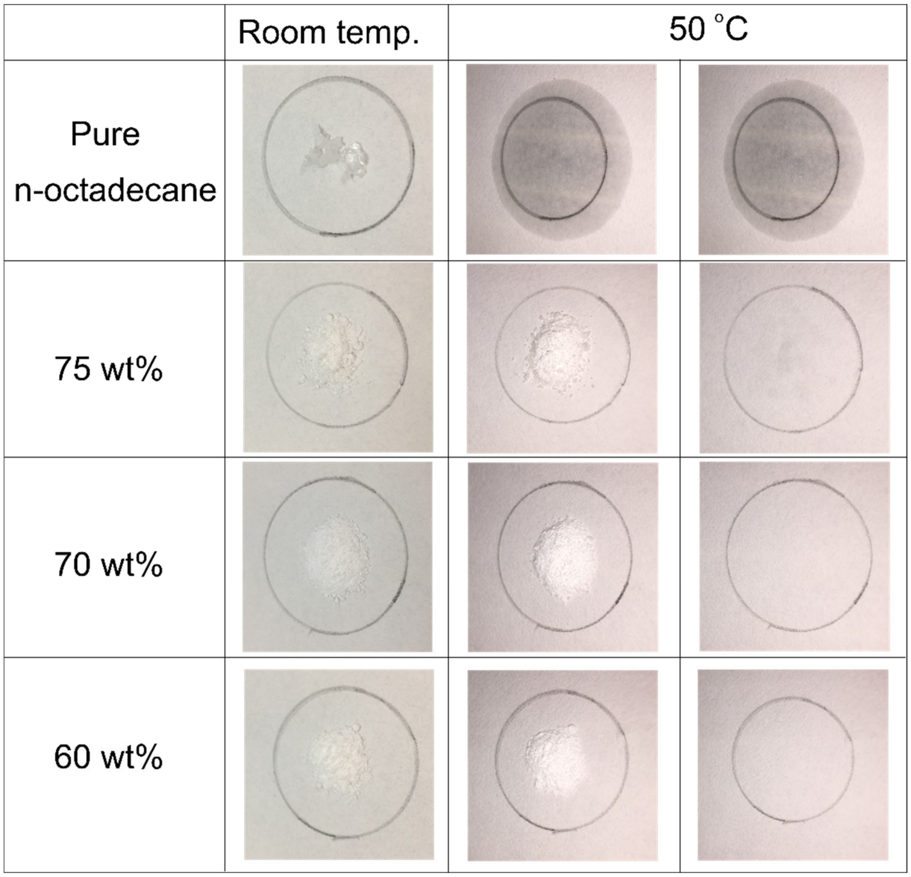

Figure S2. Photographs of pure n-octadecane and PCCs with various n-octadecane mass fractions in the leakage test.

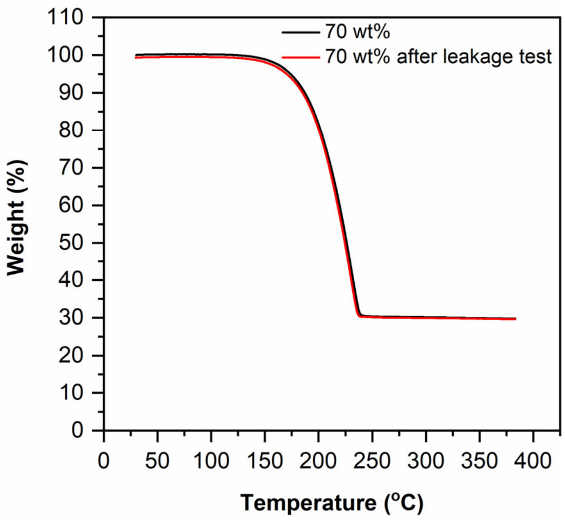

Figure S3. TGA curves of 70 wt% n-octadecane/FS PCC before and after the leakage test.
